# Comparison of manual and semi-automated delineation of regions of interest for radioligand PET imaging analysis

**DOI:** 10.1186/1471-2385-7-2

**Published:** 2007-01-29

**Authors:** Tiffany W Chow, Shinichiro Takeshita, Kie Honjo, Christina E Pataky, Peggy L St Jacques, Maggie L Kusano, Curtis B Caldwell, Joel Ramirez, Sandra Black, Nicolaas PLG Verhoeff

**Affiliations:** 1 Rotman Research Institute, Baycrest, Toronto, Canada; 2 Dept. of Medicine, University of Toronto, Toronto, Canada; 3 Dept. of Psychiatry, University of Toronto, Toronto, Canada; 4 Department of Neurosurgery, Hiroshima Red Cross Hospital & Atomic-bomb Survivors Hospital, Hiroshima, Japan; 5 Kunin-Lunenfeld Applied Research Unit, Baycrest, Toronto, Canada; 6 Dept. of Psychology, Duke University, Durham, USA; 7 Departments of Medical Imaging and Medical Biophysics, Department of Medical Physics, University of Toronto and Sunnybrook Health Sciences Centre, Toronto, Canada; 8 Imaging Research, Sunnybrook Health Sciences Centre, Toronto, Canada; 9 Cognitive Neurology Research Unit, Sunnybrook Health Sciences Centre, Toronto, Canada

## Abstract

**Background:**

As imaging centers produce higher resolution research scans, the number of man-hours required to process regional data has become a major concern. Comparison of automated vs. manual methodology has not been reported for functional imaging. We explored validation of using automation to delineate regions of interest on positron emission tomography (PET) scans. The purpose of this study was to ascertain improvements in image processing time and reproducibility of a semi-automated brain region extraction (SABRE) method over manual delineation of regions of interest (ROIs).

**Methods:**

We compared 2 sets of partial volume corrected serotonin 1a receptor binding potentials (BPs) resulting from manual vs. semi-automated methods. BPs were obtained from subjects meeting consensus criteria for frontotemporal degeneration and from age- and gender-matched healthy controls. Two trained raters provided each set of data to conduct comparisons of inter-rater mean image processing time, rank order of BPs for 9 PET scans, intra- and inter-rater intraclass correlation coefficients (ICC), repeatability coefficients (RC), percentages of the average parameter value (RM%), and effect sizes of either method.

**Results:**

SABRE saved approximately 3 hours of processing time per PET subject over manual delineation (p < .001). Quality of the SABRE BP results was preserved relative to the rank order of subjects by manual methods. Intra- and inter-rater ICC were high (>0.8) for both methods. RC and RM% were lower for the manual method across all ROIs, indicating less intra-rater variance across PET subjects' BPs.

**Conclusion:**

SABRE demonstrated significant time savings and no significant difference in reproducibility over manual methods, justifying the use of SABRE in serotonin 1a receptor radioligand PET imaging analysis. This implies that semi-automated ROI delineation is a valid methodology for future PET imaging analysis.

## Background

Advances in functional neuroimaging techniques have allowed the correlation of regions of interest (ROIs) with behavioral and cognitive tasks. Manual delineation of ROIs by trained operators is still considered the "gold standard," given its precision for the targets; however some drawbacks of manual analysis have recently been pointed out, such as its labor-intensive requirements (i.e., extensive time needed for ROI drawing) [1], limited reproducibility [2], and difficulties in measuring cortical ROIs [3]. In order to resolve these problems, some researchers have suggested other methods of analysis as represented by an automated program to label brain regions [4], automated evaluation of the whole brain [5], and automated voxel-based morphometry [6]. Unfortunately, these alternatives also are limited by ROIs available [4,5] and the potential inaccuracy introduced by spatial normalization of the brain [7]. The semiautomatic brain region extraction (SABRE) method was designed by Dade et al. to minimize the errors of both manual and automated analysis [1].

SABRE combines manual and automated analyses, which maximizes the advantages of both methods by manual definition of the most essential landmarks to create a customized atlas for the individual brain and automatic brain parcellation. SABRE has proven reliable in assessing regional tissue volume, and it provides time savings over purely manual methods.

The present study compares the benefits of the SABRE method to manual ROI delineation. We searched Pubmed for similar studies using the search terms: "automated brain region extraction," "brain region extraction," "manual ROI AND automated," "region of interest delineation," "SABRE," "semiautomated brain region extraction," and "semiautomatic brain region extraction." This yielded 491 citations. Of these, 5 described research questions similar to ours [8-11]. Three studies reported the effects of semi-automated methods vs. manual delineation methods for structural or volumetric MRI results for limited regions of brain such as hippocampus [10,11] or ventricular cerebrospinal fluid volume [9]. One of the hippocampal studies required manual delineation on the subject's first MRI, then used automated algorithms to gauge longitudinal volumetric changes from the original, individualized template [11]; the other hippocampal study used a novel expanding seed voxel with constraint points to identify 3D volumes of interest from the inside out [10]. Mosconi et al. validated automated voxel-based FDG-PET analysis including spatial normalization of hippocampal probability ROIs [12]. Only Mega et al. described a parcellation of brain into cortical regions as SABRE does [8]. Their sample also included subjects with cortical atrophy due to neurodenegerative processes but the imaging process requires warping to a standardized volumetric brainspace. Studies comparing ROI extraction reported positive conclusions in favor of using automation to save time [8,10,11] or achieving similar accuracy to manual methods [8-11], but none of them have validated the use of semi-automated methods to process functional imaging data or to process multiple cortical regions without warping. During the revision of this manuscript for publication, a paper describing a fully automated ROI extraction for use with PET imaging was published by Rusjan et al. [35]. The authors devised a fully automated method which showed time savings over manual methods and very high intraclass correlation between the two methods for use with three different radioligands. This method does not allow for individualization of intracranial capacity as in SABRE, which will be discussed below.

This study is a first time application of SABRE to a positron emission tomography (PET) study of patients with frontotemporal degeneration (FTD). As PET scanners evolve to yield larger numbers of image slices, the man-hours required to delineate ROIs for each subject become impractical. We wished to validate the use of SABRE in analyzing our PET data. Assuming that manual analysis is the gold standard, we compared the PET results generated by manual ROI drawing to those by SABRE, on the bases of analysis time, effect of analysis method on PET results, reproducibility, and ability to discriminate FTD patients from healthy control subjects. We hypothesized that SABRE would save image processing time without altering the basic quality of PET results but that SABRE would be less sensitive to detect the differences between an FTD patient group and an age-matched healthy comparison group. We also hypothesized that SABRE's test-retest reproducibility would be superior to the manual method, which might compensate for any loss of sensitivity. Balancing these characteristics might allow investigators to choose the more feasible and statistically useful procedure for future PET analyses of an FTD population.

## Methods

### Participants

We used data from 9 participants in a study comparing serotonin 1a receptor (5-HT1aR) density as estimated by radioligand binding potentials (BPs) from PET imaging data [13]. We studied 5 patients with FTD diagnosed by consensus criteria [14], duration 3–6 years. They were 1 man and 4 women, ages ranging 59–79 years, with MMSE scores 16–30, and CDR scores of 0.5). We also studied 4 age- and gender-matched healthy comparison subjects (1 man and 3 women, age range 63–80 years). The study procedures were reviewed and approved by Research Ethics Boards at all participating institutions. All 9 subjects or their substitute decision makers gave informed consent to participate in the study.

### MRI data acquisition

Imaging procedures: We conducted structural MR imaging on a 1.5 T Signa research-dedicated scanner (GE Medical Systems, software v. 8.4M4, with CV 40 mT/m gradients) at Sunnybrook Health Sciences Centre. We acquired a high-resolution T1-weighted image (an axial 3D SPGR with 5 ms TE, 35 ms TR, 1 NEX, 35° flip angle, 22 × 16.5 cm FOV, 0.859 × 0.859 mm in-plane resolution, and 1.2 to 1.4 mm slice thickness depending on head size). This was followed by an interleaved proton density (PD) and T2-weighted image set (an interleaved axial spin echo with TEs of 30 and 80 ms, 3s TR, 0.5 NEX, 20 × 20 cm FOV, 0.781 × 0.781 mm in-plane resolution, and 3 mm slice thickness). The T1-weighted and PD/T2-weighted imaging parameters have been selected to provide optimal intensity separation and are routinely used for tissue segmentation [15].

### Serotonin 1a receptor (5-HT1aR) PET acquisition

PET scans with the radioligand [^11^C]WAY-100635, a 5-HT1aR antagonist, were performed within 3 months of the MRI scans. Specific activity at time of intravenous injection of the radioligand averaged 793 ± 373 mCi/μmol. PET images were acquired for 15 transaxial slices (slice thickness of 6.5 mm) over 90 minutes with a GE Medical System PC-2048-15B camera with 5.5 mm intrinsic resolution FWHM.

Co-registered MR and PET images were used for semi-automated and manual ROI delineation, as described below.

### Manual region of interest delineation

We co-registered T1 MR images to the summed PET frames with Rview software [16], then hand-drew ROIs manually on the co-registered T1 images with the Alice™ software (Perceptive Informatics, Waltham, Massachusetts), based on previously defined landmarks (refer to Appendix I, see Additional file 1) [17-22]. Based on the proximity to the SABRE ROIs, the following twelve manual ROIs were used to generate the TACs of brain uptake: frontal lobe, orbitofrontal cortex (OFC), dorsolateral prefrontal cortex (DLPFC), anterior lateral temporal lobe (ALT), and middle amygdala-anterior parahippocampus (medial temporal) (10 ROIs across both hemispheres). Two well-trained technicians, CP (Rater M1) and PSJ (Rater M2), hand-drew ROIs.

Cortical atrophy challenges the accurate interpretation of functional images from patients with dementia. A partial volume correction (PVC) method has been adapted to correct WAY-PET imaging resolution issues. The PVC algorithm corrects for atrophy, spill-in effects, and spill-out effects. This is a variation of the algorithm by Bencherif et al. [23], modified so that calculations are performed in higher-resolution MR space. The algorithm was used to create a map of gray matter (GM) vs. non-GM pixels for each subject. We then applied the hand-drawn ROIs to the map and used Alice™ to calculate average correction factors for each ROI. We submitted the initially derived BPs to their corresponding correction factors and report here the corrected BPs.

### SABRE ROI delineation

The SABRE method uses a robust tissue segmentation protocol, which accounts for regional field (RF) inhomogeneities, noise, and partial volume effects [15]. When tested using the Montreal Neurological Imaging phantom, the coefficient of total agreement with increased noise and RF inhomogenity levels was 0.97. When tested on young normal controls and elderly Alzheimer's disease patients, the maximal differences were less than 1% of total intracranial capacity in all tissue classes in a scan-rescan test.

The SABRE process begins with segmentation of the MRI data into GM, white matter, ventricular cerebral spinal fluid and subdural/sulcal CSF (ssCSF) [1,15]. In fact, these segmentation data were used for the PVC algorithm described above. First, the operator subtracted the non-brain tissue (e.g., skull) from the T1 MR images to extract the T1 intracranial cavity (T1 eroded images). Identification of 15 landmarks on the 3D-rendered T1 images (e.g., anterior commissure (AC), central sulcus) with ANALYZE software (Biomedical Imaging Resource, Mayo Clinic, Rochester, Minnesota) yields a proportional Talairach grid of each individual's eroded T1 images [1]. Using the resulting proportional grid and defined landmarks, the SABRE program parcellates the eroded T1 images automatically into 26 zones (13 in each hemisphere).

**Figure 1:**
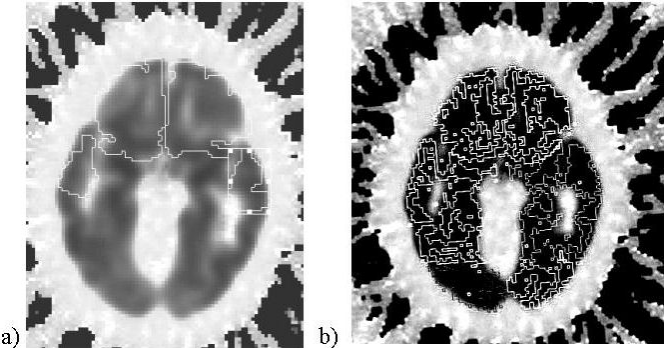
Region of interest delineation: a) manual, b) using SABRE.

To convert the SABRE zones into ROIs, we used AIR (version 5.5) to yield the optimal matrix for co-registration of the T1 masked images to the summed 0–90 minute PET frames (15 transaxial slices) [24]. We restricted the SABRE zones to GM-only portions, outlining them automatically with ANALYZE, because SABRE zones must be converted from opaque square fields to ALICE-compatible outlines. See figure 1 for an illustration of manual vs. SABRE-generated outlines of ROIs.

The same SABRE ROIs were applied to the grey matter vs. non-GM PET data map to derive SABRE-specific average correction factors for each ROI. These were then applied to the TAC data to derive the second set of partial volume corrected BPs for comparison to the manual ROI data. These procedures were performed by a neurosurgeon (ST, Rater S1) and a highly trained technician (JR, Rater S2).

### Derivation of binding potential (BP)

Manual and SABRE ROIs became the overlays applied to the dynamic PET images to calculate BP values of each ROI with Alice and PKIN/PMOD software (PMOD group, Zürich, Switzerland) [25,26]. A simplified reference tissue method (SRTM) was performed to obtain BP values, using the cerebellum as the input function [27], given previous findings that the cerebellum is relatively devoid of 5HT1aR [28] and that this method has been proven to be superior to kinetic modeling using arterial data [29].

### Statistics

Because the BP itself is a relative estimation, and the SABRE regions are inexact proxies of the hand-drawn ROIs, we did not seek direct correlations between BPs from manual vs. SABRE ROIs. Instead, we compared the methods with regard to: 1) image processing time, 2) basic quality of PET results, 3) reproducibility of BP results, and 4) sensitivity to differentiate FTD patients from comparison subjects based on BP results.

Manual raters (M1 and M2) and SABRE raters (S1 and S2) delineated ROIs on the imaging data a total of 6 times. Raters M1 and S1 repeated the process to yield the following sets of 5HT1aR BP values: M1A, M1B, M2; S1A, S1B, S2.

We averaged inter-rater processing times for each method (i.e., (M1A +M2)/2 and (S1A +S2)/2), then compared the mean time spent to process the data using either manual or SABRE methods with the unpaired Student's t-test, as we expected SABRE to save time.

We evaluated the basic quality of PET results by calculating the over all rank order of BPs among 9 subjects in each ROI with the Spearman rank correlation test. We calculated ratios of BPs in the left regions to those of the corresponding right regions (L/R ratios) for comparison of the two methods with the Wilcoxon signed rank test.

We assessed the reproducibility of the tested analysis methods based on intra- and inter-rater reliability for the BPs. We rated intra-rater reliability using intraclass correlation coefficients (ICC), repeatability coefficients (RC), and percentages of the average parameter value (RM%, a measure of coefficient of variation of the difference between the methods). ICC were based on the 1^st ^and 2^nd ^results generated by the same rater (M1A vs. M1B and S1A vs. S1B). To determine RM%, we first calculated the RC as twice the standard deviation (SD) of the difference between the average BP values for each of the ROIs from the 1^st ^and 2^nd ^analyses (e.g., M1A and M1B), expecting 95% of the differences to be less than the RC [30]. In addition, to facilitate comparisons across regions, the RC was calculated as percentage of the mean BP to obtain the RM% [27]:

RM%=2×SD(BPscan1−BPscan2)Mean(BPscan1+BPscan2)×100%
 MathType@MTEF@5@5@+=feaafiart1ev1aaatCvAUfKttLearuWrP9MDH5MBPbIqV92AaeXatLxBI9gBaebbnrfifHhDYfgasaacH8akY=wiFfYdH8Gipec8Eeeu0xXdbba9frFj0=OqFfea0dXdd9vqai=hGuQ8kuc9pgc9s8qqaq=dirpe0xb9q8qiLsFr0=vr0=vr0dc8meaabaqaciaacaGaaeqabaqabeGadaaakeaacqWGsbGucqWGnbqtcqGGLaqjcqGH9aqpdaWcaaqaaiabikdaYiabgEna0kabdofatjabdseaejabcIcaOiabdkeacjabdcfaqjabdohaZjabdogaJjabdggaHjabd6gaUjabigdaXiabgkHiTiabdkeacjabdcfaqjabdohaZjabdogaJjabdggaHjabd6gaUjabikdaYiabcMcaPaqaaiabd2eanjabdwgaLjabdggaHjabd6gaUjabcIcaOiabdkeacjabdcfaqjabdohaZjabdogaJjabdggaHjabd6gaUjabigdaXiabgUcaRiabdkeacjabdcfaqjabdohaZjabdogaJjabdggaHjabd6gaUjabikdaYiabcMcaPaaacqGHxdaTcqaIXaqmcqaIWaamcqaIWaamcqGGLaqjaaa@688F@

Inter-rater reliability was assessed by calculating ICC between manual results M1A vs. M2 and SABRE results from S1A vs. S2. We used SPSS: Analysis: Scale: Reliability: Statistics – ICC to make these calculations. We used the Wilcoxon signed rank test to compare the resulting average ICC for manual vs. SABRE results.

### Sensitivity to differentiate FTDs from controls

We used two indicators to assess the ability of the methods to differentiate FTD patients from healthy comparison subjects. At autopsy, FTD patients have significant reductions in serotonin receptor densities [31,32]; we expected to find similar losses, reflected as lower BPs, during the course of illness. First we compared the mean BPs for each ROI with paired t-tests. We also defined Cohen's measures for the effect size (*d*) as the average BP value for the FTD group minus that for comparison subjects divided by the standard deviation for the pooled samples [33]. We compared the calculated *d *values between manual and SABRE methods with the Wilcoxon signed rank test.

We used SPSS (version 15.0, SPSS, Inc., Chicago, Illinois) for all statistical analyses.

## Results

Table 1 shows the range of mean 5-HT1a R BP values. Mean BPs after partial volume correction were similar between manual and SABRE methods, without significant differences between FTD and control BPs.

**Table 1:** Average regional 5-HT1a receptor BP values

	**Manual**											
	**Patients**						**Comparison Subjects**					

	M1A		M1B		M2		M1A		M1B		M2	
	
	Lt	Rt	Lt	Rt	Lt	Rt	Lt	Rt	Lt	Rt	Lt	Rt

Frontal lobe	3.64 ± 1.89	3.57 ± 1.91	3.81 ± 2.11	3.71 ± 2.10	4.96 ± 2.07	4.96 ± 2.27	3.49 ± 0.93	3.47 ± 0.64	3.53 ± 0.86	3.47 ± 0.64	5.52 ± 1.46§	4.98 ± 1.00
OFC	5.01 ± 3.92	4.34 ± 4.35	4.89 ± 3.95	4.36 ± 4.47	5.62 ± 3.07	5.01 ± 3.43	5.38 ± 0.95	5.92 ± 1.12	5.52 ± 1.14	5.86 ± 1.11	6.12 ± 1.05	6.47 ± 1.12
DLPFC	3.87 ± 1.77	3.89 ± 1.96	3.77 ± 1.87	3.80 ± 2.06	4.70 ± 1.91	4.92 ± 2.17	3.92 ± 1.10	3.79 ± 0.61	3.78 ± 1.07	3.64 ± 0.70	4.34 ± 0.94	4.48 ± 0.87
Ant. Lat. Temporal lobe	5.25 ± 1.37	5.81 ± 2.23	5.09 ± 1.55	5.47 ± 2.39	7.58 ± 2.92	7.36 ± 1.98	5.52 ± 1.12	5.93 ± 1.27	5.37 ± 1.36	5.62 ± 1.02	5.97 ± 1.17	6.18 ± 1.02
Medial Temporal	4.58 ± 2.35	5.04 ± 2.71	4.88 ± 1.98	4.98 ± 2.64	4.97 ± 2.69	5.20 ± 2.57	4.61 ± 1.85	5.42 ± 1.25	4.48 ± 1.65	5.38 ± 1.36	5.58 ± 2.30	6.15 ± 1.90

**Average**	4.47	4.53	4.49	4.46	5.57	5.49	4.58	4.91	4.54	4.79	5.51	5.65

	**SABRE**											

	**Patients**						**Comparison Subjects**					

	S1A		S1B		S2		S1A		S1B		S2	
	
	Lt	Rt	Lt	Rt	Lt	Rt	Lt	Rt	Lt	Rt	Lt	Rt

Frontal lobe	4.09 ± 1.68	3.83 ± 1.79	3.85 ± 2.27	3.61 ± 2.36	3.99 ± 1.60	3.71 ± 1.77	4.67 ± 1.29	4.58 ± 1.24	4.55 ± 1.00	4.47 ± 0.94	4.49 ± 1.14	4.43 ± 1.10
OFC	4.07 ± 2.57	3.54 ± 2.73	3.91 ± 3.06	3.45 ± 3.18	4.01 ± 2.59	3.26 ± 2.94	5.50 ± 1.01	4.86 ± 1.03	5.57 ± 0.83	5.03 ± 0.98	5.23 ± 0.86	5.19 ± 1.39
DLPFC	4.04 ± 1.46	3.90 ± 1.48	3.78 ± 2.04	3.62 ± 2.04	4.73 ± 1.44	4.09 ± 1.54	4.27 ± 1.30	4.34 ± 1.01	4.32 ± 1.19	4.44 ± 0.96	5.61 ± 2.91	5.96 ± 2.94
Ant. Lat. Temporal lobe	5.07 ± 1.63	5.06 ± 1.61	4.74 ± 2.16	4.74 ± 2.22	4.89 ± 1.44	4.90 ± 1.50	5.42 ± 1.37	5.36 ± 1.28	5.25 ± 1.02	5.18 ± 0.89	5.27 ± 1.21	5.10 ± 1.05
Medial Temporal	5.32 ± 3.19	5.82 ± 1.98	5.61 ± 2.49	5.39 ± 2.44	5.68 ± 1.89	5.62 ± 1.83	5.95 ± 1.13	5.85 ± 0.64	6.11 ± 0.89	6.14 ± 0.66	9.19 ± 1.16	6.00 ± 0.90

**Average**	4.52	4.43	4.38	4.16	4.59	4.31	5.16	5.00	5.16	5.05	5.36	5.34

§indicates average ± SD

### Imagine processing time

The image processing time for SABRE had statistically and practically significant savings over the manual method (p < 0.0001): S1A/S2 1.2 ± .08 hours per subject vs. M1A/M2 5 ± 0.75 hours per subject; S1B/S2 1.2 ± .08 hours vs. M1B/M2 3.5 ± 0.3 hours.

### Basic quality of PET results

We found significant positive correlations between the rank order of BPs among the 9 tested subjects in the majority of rater × method comparisons, but measurements for anterior lateral and medial temporal regions were less similar (see Table 2).

**Table 2:** Correlations between 5-HT1aR BP values from manual versus SABRE ROI analysis (Spearman r_s _values) were statistically significant (p < .05) except where noted.

	**S1A vs M1A**		**S1A vs M1B**		**S1A vs M2**		**Row Average**	
	Lt	Rt	Lt	Rt	Lt	Rt	**Lt**	**Rt**

Frontal lobe	0.80	0.93	0.90	0.88	0.75	0.97	0.82	0.93
OFC	0.82	0.93	0.83	0.93	0.78	0.87	0.81	0.91
DLPFC	0.87	0.88	0.88	0.78	0.85	0.67	0.87	0.78
Ant. Lat. Temporal lobe	0.95	0.90	0.93	0.82	0.63	0.67	0.84	0.79
Medial Temporal	0.58	0.85	0.60	0.80	0.68	**0.52**†	0.62	0.72
**Column Average**	0.80	0.90	0.83	0.84	0.74	0.74	0.79	0.83

	**S1B vs M1A**		**S1B vs M1B**		**S1B vs M2**			

	Lt	Rt	Lt	Rt	Lt	Rt		

Frontal lobe	0.82	0.90	0.95	0.85	0.75	0.95	0.84	0.90
OFC	0.82	0.80	0.83	0.80	0.78	0.88	0.81	0.83
DLPFC	0.92	0.85	0.93	0.85	0.88	0.78	0.91	0.83
Ant. Lat. Temporal lobe	0.90	0.95	0.88	0.87	0.70	**0.53**†	0.83	0.78
Medial Temporal	**0.42**†	0.85	**0.33**†	0.83	**0.50**†	0.72	0.42	0.80
**Column Average**	0.77	0.87	0.79	0.84	0.72	0.77	0.76	0.83

	**S2 vs M1A**		**S2 vs M1B**		**S2 vs M2**			

	Lt	Rt	Lt	Rt	Lt	Rt		

Frontal lobe	0.80	0.93	0.90	0.88	0.75	0.97	0.82	0.93
OFC	0.97	0.80	0.98	0.80	0.93	0.88	0.96	0.83
DLPFC	0.88	0.72	0.78	0.72	0.83	0.92	0.83	0.78
Ant. Lat. Temporal lobe	0.98	0.95	0.92	0.92	0.65	**0.43**†	0.85	0.77
Medial Temporal	0.37†	0.85	**0.33**†	0.83	**0.47**†	0.72	0.39	0.80
**Column Average**	0.80	0.85	0.78	0.83	0.73	0.78	0.77	0.82

† indicates p value for correlation between manual and SABRE analysis ≥ .05

In the comparison of L/R ratios of BPs, the orbitofrontal cortex (OFC) showed the highest average L/R ratio in both manual and SABRE results for all 6 raters' measurements of BPs (see Table 3). One subject had a very small right OFC, which led to higher variance in both manual and SABRE measurements. When this subject's data were excluded, the SABRE average for raters S1A, S1B, and S2 were more similar at 1.16, 1.15, and 1.13, respectively. Most L/R ratios were very close to 1.0. After excluding the outlier, there were no statistically significant Wilcoxon results.

**Table 3:** Summary of average left-right ratios of 5-HT1aR BP values

	**Manual**		
	**M1A**	**M1B**	**M2**

Frontal lobe	1.02	1.03	1.06
OFC	1.31	1.37	**1.10**†
DLPFC	1.04	1.04	0.97
Ant Lat Temporal Lobe	0.94	0.97	0.95
Medial Temporal	0.91	0.97	0.92

	**SABRE**		

	**S1A**	**S1B**	**S2**

Frontal lobe	1.04	1.04	1.06
OFC	1.22	1.32	2.96
DLPFC	1.02	1.02	1.03
Ant Lat Temporal Lobe	1.01	1.01	1.01
Medial Temporal	0.90	1.02	1.02

OFC = orbitofrontal cortex, DLPFC = dorsolateral prefrontal cortex, Ant Lat Temporal

Lobe = anterior lateral temporal lobe

† Wilcoxon p < 0.05 vs. S1A

### Reproducibility of BP results

SABRE methods achieved average intra-rater (S1A vs. S1B) ICC values similar to the manual methods (see Table 4), but Wilcoxon rank testing showed significant differences in average RC and RM%, supporting manual methods as more reliable when examining intra-rater performance. As shown in the table, the RM% had a wide range across ROIs.

**Table 4:** Intra-rater reliability of intra-class correlation coefficients and repeatability coefficients

	**Manual (M1A-M1B)**					
	Lt			Rt		

	ICC	RC	RM %	ICC	RC	RM %

Frontal lobe	0.99	0.46	6.33	1.00	0.43	6.02
OFC	1.00	0.73	6.92	1.00	0.86	7.51
DLPFC	1.00	0.68	6.58	0.99	0.43	4.31
Ant Lat Temporal lobe	0.98	0.35	4.52	0.98	0.46	6.11
Medial Temporal	0.98	1.09	11.74	1.00	0.34	3.31
**Average**	**0.99**	**0.66**	**7.22**	**0.99**	**0.51**	**5.45**

	**SABRE (S1A-S1B)**					

	Lt			Rt		

	ICC	RC	RM %	ICC	RC	RM %

Frontal lobe	0.99	1.07	12.57	1.00	1.03	12.57
OFC	1.00	0.91	9.75	1.00	0.87	10.45
DLPFC	0.99	1.15	14.12	1.00	1.23	15.25
Ant Lat Temporal lobe	0.99	1.27	12.46	0.99	1.38	13.60
Medial Temporal	0.99	2.38	20.82	0.99	1.85	16.01
**Average**	**0.99**	1.36*	13.94*	**0.99**	1.27*	13.58*

OFC = orbitofrontal cortex, DLPFC = dorsolateral prefrontal cortex, Ant Lat Temporal

Lobe = anterior lateral temporal lobe

* p = 0.043 vs. manual results, Wilcoxon rank test

SABRE results yielded high ICC values for inter-rater reliability in general (see Table 5). Average SABRE ICC ranged from 0.91 – 0.97 on both hemispheres, with lower ICC for DLPFC ROIs. Rater S2's BP results for DLPFC from one control were exceptionally large (~9.5 vs. ~5). When the data for this subject were excluded, ICC for this ROI increased to 0.83, 0.79, 0.85, and 0.85, reading left to right across Table 5.

**Table 5:** Inter-rater reliability intra-class correlation coefficients

	**Manual**			
	**M1A vs M2**		**M1B vs M2**	

	Lt	Rt	Lt	Rt

Frontal lobe	0.94	0.96	0.93	0.96
OFC	0.96	0.95	0.96	0.95
DLPFC	0.95	0.97	0.95	0.97
Ant Lat Temporal Lobe	0.50	0.62	0.57	0.67
Medial Temporal	0.98	0.98	0.94	0.99
**Average**	**0.87**	**0.90**	**0.87**	**0.91**

	**SABRE**			

	**S1A vs S2**		**S1B vs S2**	

	Lt	Rt	Lt	Rt

Frontal lobe	0.99	1	0.96	0.97
OFC	0.99	0.98	0.98	0.99
DLPFC	0.69	0.65	0.97	0.78
Ant Lat Temporal Lobe	0.99	0.98	0.95	0.94
Medial Temporal	0.92	0.96	0.97	0.94
**Average**	**0.92**	**0.91**	**0.97**	**0.92**

OFC = orbitofrontal cortex, DLPFC = dorsolateral prefrontal cortex, Ant Lat Temporal Lobe = anterior lateral temporal lobe

In comparison, ICC values for the manual method were slightly lower averages, ranging 0.79–0.87. As opposed to the DLPFC, the lowest ICC for the manual ratings were in the anterior lateral temporal lobe. No cross-method comparisons were statistically significantly different.

### Sensitivity to differentiate FTD patients from healthy comparison subjects

Average BP values of FTD patients did not differ from those of healthy comparison subjects according to the unpaired Student's t-test, regardless of the method used to delineate ROIs. Cohen's *d *values (effect sizes) for the SABRE method were higher than for the manual method across all ROIs (see Table 6). SABRE-derived *d*'s exceeded manually-derived *d's *with p < 0.05, except in the comparison against rater M2. This particular finding supports the lower inter-rater reliability of the manual method. Effect sizes for left and right OFC were larger than for other ROIs. As in Table 3, the right OFC BP from one subject was thought to be an outlier. Values for right OFC effect sizes when this subject was excluded still varied greatly, reflecting the difficulty of measuring BP when the ROI is very small: M1A 0.24, M1B -0.19, M2 -0.28, S1A -0.37, S1B -0.39, and S2 -0.55.

**Table 6:** Cohen's measure for effect size using manual versus SABRE analysis to differentiate FTD from controls

	**Manual**					
	M1A		M1B		M2	

	Lt	Rt	Lt	Rt	Lt	Rt

Frontal lobe	0.11	0.08	0.19	0.18	-0.32	-0.01
OFC	-0.15	-0.58	-0.25	-0.54	-0.24	-0.64
DLPFC	-0.04	0.08	-0.01	0.12	0.25	0.29
Ant Lat Temporal Lobe	-0.22	-0.06	-0.20	-0.09	0.74	0.71
Medial Temporal	-0.01	-0.19	0.22	-0.20	-0.24	-0.42
**Average**	**-0.06**	**-0.13**	**-0.01**	**-0.11**	**-0.26**	**-0.30**

	**SABRE**					

	S1A		S1B		S2	

	Lt	Rt	Lt	Rt	Lt	Rt

Frontal lobe	-0.39	-0.49	-0.43	-0.52	-0.37	-0.50
OFC	-0.80	-0.70	-0.85	-0.76	-0.71	-0.89
DLPFC	-0.17	-0.36	-0.34	-0.54	-0.57	-0.84
Ant Lat Temporal Lobe	-0.24	-0.20	-0.32	-0.28	-0.29	-0.15
Medial Temporal	-0.29	-0.02	-0.29	-0.49	-0.33	-0.28
**Average**	-0.38*	**-0.35**	-0.45*	**-0.52**†	-0.45*	**-0.53**†

OFC = orbitofrontal cortex, DLPFC = dorsolateral prefrontal cortex, Ant Lat Temporal

Lobe = anterior lateral temporal lobe

* p < 0.05 vs. left M1A & M1B

† p < 0.05 vs. right M1A & M1B

## Discussion

Prior studies have shown that automated methods of ROI delineation can be accurate and time-saving for structural volumetric analyses [8-11]. Our present results indicate that the SABRE method also saves time for functional radioligand PET analysis without altering the basic quality of the results as compared to the gold standard, manual ROI analysis. Intra-rater ICC and reliability were greater for manual methods than SABRE, exceeding reliability criteria pegging acceptable ICC values at a range of 0.75–0.80 [34]. Inter-rater ICC also met acceptable ICC value criteria, with the exception of manual anterior lateral temporal ROIs. The inter-rater reproducibility of PET results using SABRE was at least as high as that using the manual method. Although SABRE failed to significantly discriminate FTD patients from healthy comparison subjects, which may be related to the small sample size, higher *d *values for SABRE imply that SABRE can detect the expected 5-HT1a R BP differences between FTD patients and comparison subjects more sensitively than manual analysis.

The image processing time savings are amplified for datasets where more than 15 slices are available: compared to more current scanners that would afford 124 slices, our approximately 3 hour difference between methods would translate to at least an 8-hour saving (2 hours for SABRE vs. at least a 10 hour manual task). 

Our results were very similar to those from the fully automated ROI extraction method of Rusjan et al. [35], except that they were able to demonstrate higher intra- and inter-rater reliability for the automated method by virtue of full automation obviating normal human variation. We would have expected the SABRE method to grant advantages over the fully automated method due to use of individualized ROI extraction instead of subjecting MRI data to a normalization procedure prior to co-registration with the PET data, but we encountered problems with reliability of SABRE in the temporal lobe regions. Both of our studies recommend automated methods as a time-saving method for ROI extraction without significant cost to accuracy.  

Limitations of this study include low sample size and difficulty pinpointing the differences between methods specific to the manual vs. SABRE aspect. Ideally, a validation study would include a larger group of imaging data, as well as more inter-rater comparisons. Using a small number may bias our search for similarity of data quality in favor of SABRE. A larger sample would make the analysis less vulnerable to outliers. Only Mega et al.'s study [8] included 20 subjects (more than twice our sample), consisting of both patients with neurodegenerative disease and controls.

Inclusion of both subjects with moderate to severe cortical atrophy due to FTD and healthy controls with little or no atrophy may have compensated for the small sample size by creating a varied landscape over which both methods had to perform, but the atrophic ROIs may have complicated reproducibility of anterior lateral temporal, DLPFC, and right OFC delineation. A further important limitation is our method of correcting for partial volume effects, in which we applied correction factors to the regional BPs and not to the individual data points along the time activity curve (TAC). Our partial volume effect method suffices for the purpose of our comparison, but most investigators will perform partial volume effects compensation at an earlier data modeling step.

Differences between the methods may be related to aspects of image processing other than the actual delineation of the ROIs. We used Rview for co-registration of the MRI to the PET images for the manually derived data and AIR for the SABRE data. Software also differed for tracing the ROIs: manual raters used Alice; SABRE raters used ANALYZE. These software variations are difficult to include as covariates in the analysis and cannot be ruled out as confounders. It would be difficult to conceive of a significant impact of the software upon the time saved in image processing.

The validation results reported here only apply to this specific experimental setup. It is not known how the accuracy of the procedure is affected by errors in MRI segmentation and/or MRI-PET coregistration, which differ when other radiotracers or segmentation and coregistration strategies are used.

Our findings that SABRE saved time over manual drawing of multiple ROIs are not surprising; the most similar studies in the literature are in agreement [8-11]. Because structural landmarks are the bases of ROIs processed in the interpretation of PET images, it seemed consistent to find that reproducibility for the SABRE method was equivalent to manual methods. The SABRE method requires identification of fewer anatomical landmarks (15), as opposed to boundaries for each of 10 ROIs in the manual process (approximately 60 localizations, see Appendix) and therefore should leave less room for variation between raters. Ashton et al.'s valid concern about error due to the tracking between slices required from 2D techniques [10] could not be evaluated in our comparison, as derivation of data for the BP measurements uses 2D techniques and would therefore be exposed to the same types of edge detection limitations.

## Conclusion

This first account of semi-automated ROI delineation improving on manual methods in processing functional neuroimaging data validates the use of SABRE for future PET studies where the analysis relies upon hypothesis-based inquiry of ROIs. Investigators are cautioned about the potential for reduced reliability using either method when studying ROIs featuring marked atrophy in patient subjects.

## Competing interests

The author(s) declare that they have no competing interests.

## Authors' contributions

TWC conceived the study. CP and PSJ recruited subjects to the study, coordinated data transfer between imaging centers, and conducted the manual ROI delineation. JR also assisted with data transfer between centers and performed SABRE analyses. ST performed SABRE analyses, participated in the design of the study, and performed the statistical analysis. CP assisted in formatting of the manuscript. KH also performed statistical analyses and assisted with figure preparation. TWC, SB, and NPLGV participated in the design of the study and crafted the manuscript. MK and CC processed the WAY-PET partial volume corrections and generated the PET data maps. All authors read and approved the final manuscript.

## Pre-publication history

The pre-publication history for this paper can be accessed here:



## Supplementary Material

Additional file 1 Appendix I. Summary of ROI definition for manual drawing. Landmark definitions for hand-drawing of manual ROIs on the co-registered T1 images using the Alice™ software (Perceptive Informatics, Waltham, Massachusetts). Click here for file
