# Supplementary material for: Comparison of manual and semi-automated delineation of regions of interest for radioligand PET imaging analysis
Source: BMC Nucl Med. 2007 Jan 29;7:2. doi: 10.1186/1471-2385-7-2 (PMC1802071; doi:10.1186/1471-2385-7-2)
Supplement: Additional file 1 — Appendix I. Summary of ROI definition for manual drawing. Landmark definitions for hand-drawing of manual ROIs on the co-registered T1 images using the Alice™ software (Perceptive Informatics, Waltham, Massachusetts). [file 1471-2385-7-2-S1.doc]

**Appendix I. Summary of ROI definition for manual drawing**

**Frontal lobe**[35]

- Superior border: Vertex
- Inferior border: Most inferior axial slice including orbitofrontal cortex (OFC)
- Antero-lateral border: Skull
- Superoposterior: Central sulcus laterally and imaginary line perpendicular to the interhemispheric fissure passing through the central suclus medially (*anterior border of the anterior cingulate gyrus in the slices where it is recognized)
- Inferoposterior: Operculum/rostral Sylvian fissure laterally and imaginary line perpendicular to interhemispheric fissure passing through anterior border of the insula medially
- Medial: Interhemispheric fissure

**Orbitofrontal Cortex (OFC)**[35]

- Superior: The olfactory sulcus is visualized in its full anterior to posterior extent (or 50% or more of it is visualized, when there is no slice with the full extent visualized).
- Inferior: Skull base
- Anterior: Frontal bone
- Posterior: Sylvian fissure cleft. Where the Sylvian fissure is not visualized medially, a line will be drawn from the inter-hemispheric fissure at the level of the optic tract medially to the Sylvian fissure laterally.
- Medial: Interhemispheric fissure
- Lateral: Frontal bone

**Dorsolateral prefrontal cortex: Brodmann areas 6, 9, 10, 44, 45 and 46[19, 36]**

- Superior to inferior extent: From above OFC to vertex[19]
- Anterior: Frontal pole[19]
- Posterior: Precentral sulcus[36]

*Postcentral sulcus: meet the intraparietal sulcus & reach Sylvian fissure

- Medial: Imaginary line, parallel to interhemispheric fissure, passing through the most lateral point of the cortex adjacent to interhemispheric fissure *(Modified from[19])*
- Lateral: Frontal bone[19]

#### Lateral anterior temporal lobe

- Superior: Sylvian fissure
- Inferior: The slice in which the middle cerebellar peduncle is visualized
- Anterior: Sylvian fissure
- Posterior: Perpendicular plane to interhemispheric fissure and canthomeatal line, which passes through the intersection of post-central sulcus to Sylvian fissure
- Medial: Occipitotemporal sulcus
- Lateral: Temporal bone

**Middle amygdala – anterior parahippocampus (MAAP)**

- Superior: Endorhinal sulcus which constitutes of suprasellar cistern[35]
- Inferior: Collateral sulcus[35]
- Anterior: Medial portion of the Sylvian (lateral) fissure (Limae insula). When the grey matter along the Sylvian fissure connects with MAAP, we will arbitrarily continue our medial border of the amygdaloid nucleus until it connects with the Sylvian fissure[17]
- Posterior: Anterior border of hippocampus which corresponds to the uncal recess of the temporal horn. Where the uncal recess is not clearly defined medially, a transverse line parallel to the uncal recess will be arbitrarily drawn. Where the uncal recess or choroidal fissure are not well visualized, the boundary between the hippocampus and MAAP will be arbitrarily drawn as a line extending laterally from the most anterior extension of the midbrain[17, 18]
- Medial: Cerebrospinal fluid within the suprasellar cistern[17]
- Lateral: Medial border line of superior temporal gyrus[35]
